# A Manual of Medical Jurisprudence

**Published:** 1866-12

**Authors:** 


For sale by S. C. Griggs & Co., Lake St., Chicago.

A Manual of Medical Jurisprudence. By Alfred Swaine Taylor, M.D., F.R.S., Fellow of the Royal College of Physicians, and Prof. of Med. Jurisprudence and Chemistry in Guy's Hospital. Sixth American, from the eighth and revised London edition. With notes and references to American decisions, by Clement B. Penrose, of the Philadelphia Bar. Philadelphia: Henry C. Lea. 1866.

This is a well-executed volume of 776 pages. The former editions have been long enough before the profession to be well known. The value of the present edition is increased by the notes of the present editor referring to legal decisions in the American Courts. While in fulness and completeness it probably cannot compare with the larger work of the late Dr. Beck, it is, nevertheless, one of the best text-books on medical jurisprudence in our language.

